# Engineering a responsive DNA triple helix into an octahedral DNA nanostructure for a reversible opening/closing switching mechanism: a computational and experimental integrated study

**DOI:** 10.1093/nar/gky857

**Published:** 2018-09-22

**Authors:** Alessio Ottaviani, Federico Iacovelli, Andrea Idili, Mattia Falconi, Francesco Ricci, Alessandro Desideri

**Affiliations:** 1 Biology Department, University of Rome Tor Vergata, Rome 00133, Italy; 2 Chemistry Department, University of Rome Tor Vergata, Rome 00133, Italy

## Abstract

We propose an experimental and simulative approach to study the effect of integrating a DNA functional device into a large-sized DNA nanostructure. We selected, as a test bed, a well-known and characterized pH-dependent clamp-switch, based on a parallel DNA triple helix, to be integrated into a truncated octahedral scaffold. We designed, simulated and experimentally characterized two different functionalized DNA nanostructures, with and without the presence of a spacer between the scaffold and the functional elements. The experimental and simulative data agree in validating the need of a spacer for the occurrence of the pH dependent switching mechanism. The system is fully reversible and the switching can be monitored several times without any perturbation, maintaining the same properties of the isolated clamp switch in solution.

## INTRODUCTION

The field of DNA nanotechnology has experienced an enormous growth in the last years, thanks to the implementation of efficient assembling strategies that allow to create nanoscale structures of increased complexity and precision (1). DNA exhibits enhanced stability, biocompatibility and full programmability, making this molecule a suitable tool to engineer nanostructures of different dimension and shape (2). In the last 30 years, different assembly methods have been developed spanning from an arrangement of few DNA tiles (3–5) to the use of thousands of short staples to fold a long single strand, known as DNA origami concept, which permits the building of complex non-arbitrary 2D and 3D structures (6–8). Several structures have been built from small two or three-dimensional arrays (3,9,10) to regular DNA polyhedra (11–14), up to the assembly of micrometre-scale DNA origami arrays with arbitrary patterns (15) or larger, gigadalton-scale DNA nanostructures (16). Another valuable strategy for the development of nanomechanical devices concerns the use of chemical modifications to build information rich, uniquely addressable DNA nanostructures (17,18). These methods allowed the introduction of modular building blocks for the construction of high yield novel nanodevices stable to heat and denaturation agents. Providing a functional utility to DNA nanostructures represents the ultimate goal for their potential use in bio-medical or bio-technological applications. In this perspective several examples have recently demonstrated the use of DNA-based structures to encapsulate and transport different payloads, like anticancer drugs such as doxorubicin (19,20) or to trap proteins (21–23) to be protected from a hostile environment and delivered into specific cells. An example is a DNA cube decorated with dendritic alkyl chains able to form a monodisperse micelle within its cavity to trap hydrophobic molecules presented by the Sleiman group (19). In order to achieve a reversible loading and release of a drug or a protein the DNA nanostructure should be functionalized with responsive elements that can undergo reversible input-induced conformational changes or confer a specific function to the, otherwise inert, structure. Different inputs can in principle be used in this regard such as small molecules, specific DNA sequences, pH, light or temperature (24,25). A DNA bipyramid with an opening mechanism triggered by UV-light has been described (26) and a similar approach has been proposed to induce an isothermal pH-induced disassembly of a tetrahedron through the reversible formation and dissociation of a triple helix (27). Triple helical motifs has been also used as functional units to address and precisely locate the assembly of nonrepetitive 2D DNA grids (28) and 3D crystals (29). Another interesting example is a DNA icosahedron able to displace its molecular cargo upon controlled opening after cdGMP molecules binding (30). The integration of functional responsive units in DNA nanostructures is, however, not straightforward. For example, we recently integrated temperature-dependent hairpins into an octahedral DNA nanocage to allow the reversible encapsulation and release of a protein (12,22). The responsive elements integrated into the DNA nanostructures showed a temperature-dependent behaviour that was significantly different to that observed in the isolated state in solution (22). A computational analysis coupled to experiments permitted us to describe the behaviour of the responsive elements in the nanostructure and to ascribe the difference to the geometrical constraints imposed by the nanostructure (12,22).

Motivated by the above results here we propose a computational and experimental integrated approach to study the effect of engineering a functional responsive DNA-based element into a DNA nanostructure. We have selected a well-known and characterized pH-dependent clamp-switch based on the formation and dissociation of a parallel DNA triple helix (31,32), and we designed a novel octahedral cage decorated with two copies of this functional element. The effect of the nanostructure environment on the pH-dependent behaviour of the responsive element has been investigated through accelerated molecular dynamics (aMD) simulation, fluorescence and gel electrophoresis. Results indicate that the presence of a spacer between the scaffold and the clamp-switch is required to retain the reversible pH-dependent mechanism observed for the isolated element in solution, confirming the importance of integrating simulative tools and experiments to rationally design functional, stimuli-responsive nanostructures.

## MATERIALS AND METHODS

### Model building of the DNA cages

**Figure 1.**
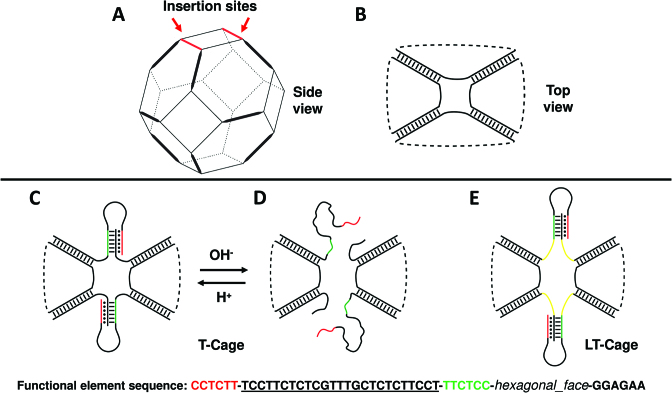
Modelling of the pH-dependent DNA nanocages. (**A**) Truncated octahedral DNA cage. Thick lines indicate double helices, thin lines the 5T linkers. (**B**) Top view of the truncated octahedral cage. (**C**) Top view of the T-cage, functionalized with two pH-dependent functional elements, at pH 5.0 and at (**D**) pH 8.0. (**E**) Model of the LT-cage, functionalized with two pH-dependent functional elements connected to the cage scaffold through seven-base spacers (yellow). The sequence of the clamp-switch triplex functional element is shown at the bottom of the figure. The green and black sequences represent the two strands interacting through the W-C interactions, the underlined sequence represents the 25 bases loop and the red sequence the third strand establishing at pH 5.0 the Hoogsteen hydrogen bonds with the double helix.

The octahedral scaffold of the DNA nanocages was built through our Polygen software (33) designing eight oligonucleotides sequences (See supplementary data, Oligonucleotide sequences section) based on those previously used to experimentally assemble different truncated octahedral geometries (34–36). The *fiber* module of the X3DNA program (37) has been used to generate the PDB file template of the triple helix model, exclusively formed by TAT sequence repetitions. The nucleotide sequence of the strands composing the triple helices has been modified through the X3DNA *mutate_bases* module (37) in order to match the designed oligonucleotides sequences. The Watson-Crick (W-C) strand has been connected to the triplex forming strand through the *sculpting* module of the PyMol program (38) to generate the triplex clamp-switch. The structure has been minimized using the UCSF Chimera program (39) to remove any clashes and unwanted interactions introduced by the modeling. The T-cage (Figure 1C) and LT-cage (Figure 1E) structures were modeled using the SYBYL 6.0 program (TRIPOS, http://www.tripos.com), manually adding two copies of the clamp-switch to the octahedral scaffold. In the case of LT-cage, two 7-thymidine spacers has been introduced (Figure 1E, yellow lines) between the double helical portion of the clamp and the cage scaffold. The steric clashes introduced by the modelling procedure have been removed through the SYBYL *anneal* module and then a first minimization of the entire structures has been performed using the SYBYL *maximin2* module. The system topologies and the coordinates of the triple helix at the two pH conditions (i.e., pH 5.0 and 8.0), used as input for the NAMD 2.12 MD package (40), have been obtained through the AmberTools tLeap module (41), parameterizing the structures through the AMBER ff14SB force field (42) with the parmbsc1 corrections (43). The structures have been immersed in a cubic box filled with TIP3P (44) water molecules, imposing a minimum distance between the solute and the box of 14 Å, whereas the charges have been neutralized adding, in electrostatic favourable positions, magnesium counter-ions to the solvated systems. To simulate the pH 5.0 conditions, the residue names of cytosines, composing the triplex-forming strand, were changed according to the AMBER nomenclature for protonated nucleotides.

### Equilibration and MD protocol

The nanocages were subjected to two minimization runs. In the first one restraints of 5.0 kcal/mol•Å^−2^ have been imposed on all the cages atoms to relax the water molecules and the ions. After, the cages have been minimized without any restraint to relax the entire system. A thermalization procedure using the NVT ensemble has been carried out on the minimized structure, gradually heating the systems from 0 to 300 K increasing temperature of 10 K every 30 ps. The optimized systems have been then simulated using periodic boundary conditions for 150 ns, with a 2.0 fs time-step, using the isobaric-isothermal ensemble (NPT). The electrostatic interactions have been calculated every 4.0 fs, using a cut-off of 10 Å for the evaluation of short-range non-bonded interactions and the PME (45) method for the long-range electrostatic interactions. The SHAKE (46) and the SETTLE (46) algorithms have been used to constrain the nucleic acids and the water molecules, respectively. Temperature has been fixed at 300 K using Langevin dynamics (47), while pressure has been held constant at 1 atm through the Langevin piston method (48). The atomic positions have been saved every 1000 steps (2.0 ps) for the analyses. The simulations have been performed using 64 nodes, for a total of 4352 CPUs, on the A2 partition of the CINECA MARCONI HPC cluster.

### Trajectory analysis

Root-mean-square deviations (RMSDs), hydrogen bonds time evolution and distance analyses have been carried out over the entire 150 ns trajectories by using the GROMACS 2016.1 analysis tools (49,50). The hydrogen bond number was evaluated, through the *hbond* module, using an angle cut-off of 30° and a donor-acceptor distance of 3.5 Å. MM/GBSA calculations were performed using the MMPBSA.py (51) code included in the AmberTools distribution (41). The solvent accessible surfaces were calculated through CPPTRAJ (52) of the AmberTools distribution, while the buried surface areas (BSA) were computed by the formula:

\begin{equation*}{\rm BSA} = \frac{{{{\rm SAS}_{{\rm system}}} - {{\rm SAS}_{{\rm clamp} - {\rm switch}}}{{\rm -SAS}_{{\rm target}}}}}{2}\end{equation*}

### Experimental assembly and characterization of the DNA nanostructures

All DNA nanocages were prepared as described (13) with some modifications. All oligonucleotides (see supplementary data, Oligonucleotide sequences section) were HPLC purified and purchased from Integrated DNA technologies (IDT) and IBA. All DNA nanocages oligonucleotides, except those including the pH-dependent motifs, were purchased phosphorylated at the 5′. The sequences of the oligonucleotides are reported in the Oligonucleotides section. Briefly, cages were assembled by combining equimolar amounts of each strand at a concentration of 1 μM in an assembly buffer containing 40 mM Tris-acetate (pH 8.0) and 15 mM MgCl_2_. Samples were heated to 95°C for 5 min then 80°C for 5 min, cooled to 60°C (4 min/1°C), and finally slowly cooled to 4°C (6 min/1°C). Cages were then incubated for 2 h at 25°C with T4 DNA ligase (New England Biolabs) to covalently close all nicks and analyzed on native 6% polyacrylamide gels in TAE buffer 1× (40 mM Tris, 2 mM EDTA adjust to pH 8.0 with acetic acid) supplemented with 10 mM MgCl_2_ for 4 h in cold buffer at 250 V. The 50 bp ladder was purchased from New England Biolabs. After staining with ethidium bromide, the band of correctly assembled cages was cut out of the gel and grounded into a fine powder, by freezing with liquid nitrogen. Three volumes of 15 mM MgCl_2_, 10 mM Tris-acetate (pH 8) was added to the powder to elute the DNA. After soaking overnight at room temperature, the residual gel powder was filtered off using a 0.45-mm filtration spin column. The samples were concentrated by using an YM-10 centrifugation column (Microcon) and then quantified by a Nano spectrophotometer measuring the double strand absorbance at 260 nm. To control the purification products 100 ng of nanocages were run on a native gel. Assembly quantification was performed using ImageJ (data not shown).

### pH-titration curves

pH titration curves were obtained using a Varian Cary Eclipse Fluorometer with excitation at 370 (±5) nm and acquisition between 405 (±5) and 650 (±5) nm at a temperature of 25°C using a total volume of 800 μl (for unimolecular clamp switch and its control probe) or 100 μl (for LT-cage and its control) in a quartz cuvette. Fisetin was dissolved in DMSO at a concentration of 500 μM and stored at −20°C. This stock solution was then diluted in the working buffer (1× TAE buffer – 40 mM Tris, 20 mM acetic acid, 1 mM EDTA) at a concentration of 500 nM. The pH of the working buffer was adjusted to the desired value using a 3 M NaOH or HCl solution. The fluorescence signal of fisetin in absence of DNA was initially recorded. After a stable signal was obtained the DNA switch or its duplex control (2 μM) were added. After 10 min, the fluorescence signal of fisetin in the presence of the relative DNA switch was recorded. The same procedure was adopted with the triplex-cage and its duplex control (0.25 μM). The pH-titration curves (Figure 6 and Supplementary Figure S5) show the difference of the fisetin fluorescence signal at 538 nm in presence and in absence of DNA switch/control or DNA cage/control for each pH value tested. Using this signal difference, the pH titration curves were fitted using the following equation:

\begin{equation*}\Delta F = \Delta {F_{{\rm Triplex}}} + \left( {\frac{{\left[ {{{\rm H}^ + }} \right] \cdot \left( {\Delta {F_{{\rm Triplex}}} - \Delta {F_{{\rm Duplex}}}} \right)}}{{\left[ {{H^ + }} \right] + \ {K_{\rm a}}}}} \right)\end{equation*}

where Δ*F*_triplex_ and Δ*F*_duplex_ represent the difference in fluorescence intensities of the fisetin in obtained at a pH of 4.5 and 8.5 respectively and where [H^+^] represents the total concentration of hydrogen ions and *K*_a_ is the observed acid constant for the switch or nanocage.

### Kinetic fluorescence experiment

The kinetic experiment (Figure 6) was performed using a Varian Cary Eclipse Fluorometer with excitation at 370 (±5) nm, acquisition at 538 (±5), average time 0.4 s and at a temperature of 25°C. Fisetin was diluted in the working buffer at pH 5.0 at a concentration of 500 nM in a 1400 μl volume stirred cuvette. The fluorescence signal of fisetin was recorded and the pH of the solution was cyclically changed between pH 8.0 and 5.0 by addition of small aliquots of 3 M NaOH or HCl. Using a similar working solution at pH 5.0 the LT-cage was diluted (0.25 μM) and the fluorescence signal of fisetin was recorded. Also in this case the pH of the solution was cyclically changed between pH 8.0 and 5.0 by addition of small aliquots of 3 M NaOH or HCl. The time-course graph shows the difference of the fisetin fluorescence signal at 538 nm in presence and absence of LT-cage.

## RESULTS

### Models of the pH-dependent DNA nanocages

As model system we employed a truncated octahedral DNA cage structure, assembled starting from eight different oligonucleotides (see Supplementary data). In this system, the edges of the octahedral geometry are represented by twelve DNA double helices (Figure 1A, thick lines), while the truncated faces are constituted by four DNA single strands composed by five thymidines (Figure 1A, thin lines). In order to provide a functionality to such cage we have re-engineered two of the eight oligonucleotides employed for the cage assembly replacing two 5T linkers (Figure 1B) with two pH-dependent DNA functional units (Figure 1C; sequence is shown below). The two strands integrate a pH-dependent triplex forming clamp-switch domain (31). One portion of the switch is formed by the W-C hybridization of two six bases complementary sequences (Figure 1C, green and black strands connected by black dashes), connected to one hexagonal face of the octahedron. The triplex-forming sequence, consisting of six bases, is connected to one of the W-C strands through a 25 bases loop specifically interacting at pH 5.0 through Hoogsteen hydrogen bonds with the double helical portion (Figure 1C, red and black strands, connected by black dots). When brought at pH 8.0, the clamp-switch domain should unfold (Figure 1D) as demonstrated for the isolated switch in solution (32). To study the effect of the octahedral scaffold on the behaviour of these functional elements, we designed two different nanocage models. In the first one, from here T-cage, the clamp-switch is directly connected to the cage scaffold as illustrated in Figure 1C. In the second system, from here LT-cage, two seven-thymidine spacers were inserted between the cage scaffold and the pH-dependent units (Figure 1E, yellow lines).

### Simulative structural/dynamical evaluation of the responsive element

**Figure 2.**
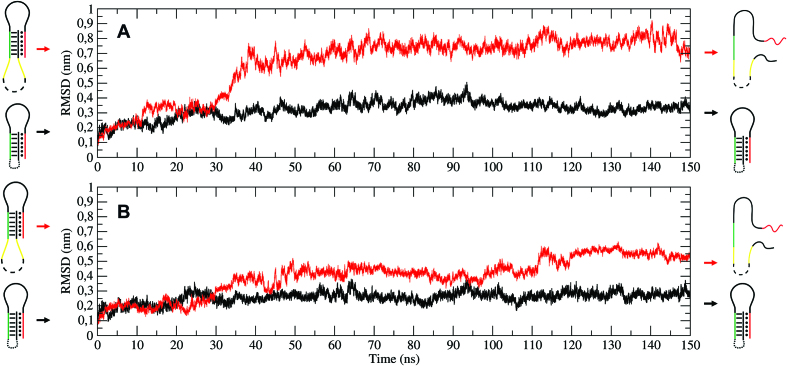
Time-dependent evolution of RMSD values. RMSD of the triple-helix (**A**) and of the double-helix region (**B**) evaluated at pH 8.0 for one of the clamp-switch element for the T-cage (black line) and the LT-cage (red line).

**Figure 3.**
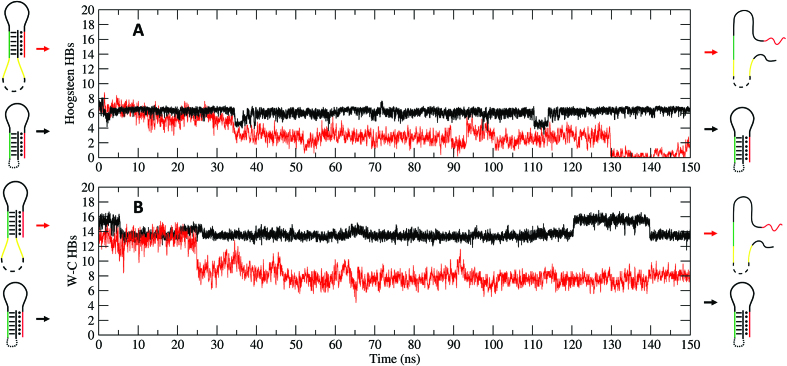
Evolution of the hydrogen bond number between the double helix and the triplex-forming strand (**A**, Hoogsteen panel) and within the double helix (**B**, Watson–Crick panel) at pH 8.0 for the T-cage (black line) and the LT-cage (red line).

**Figure 4.**
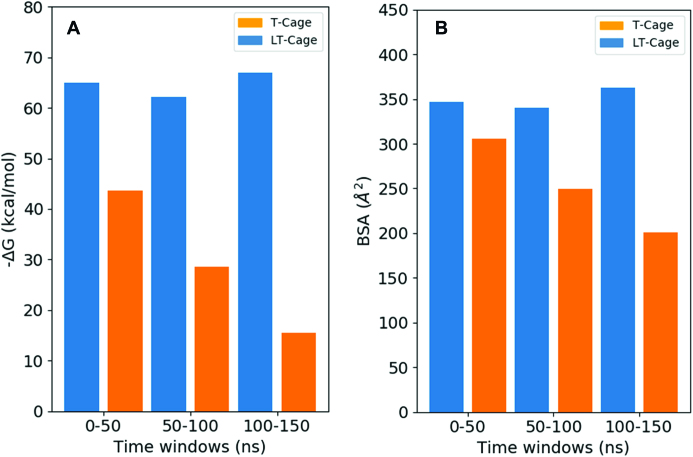
Analyses of the MM/GBSA interaction energy and of the buried surface area for the 0–50, 50–100 and 100–150 ns trajectories time windows. (**A**) MM/GBSA interaction energy at pH 8.0 for the clamp-switch of the T- (blue) and LT-cage (orange), respectively. (**B**) BSA values at pH 8.0 for the clamp-switch of the T- (blue) and LT-cage (orange).

The behaviour of the clamp-switch mechanism in the two different models has been investigated at the atomistic level, using accelerated molecular dynamics simulations. The aMD simulations have been performed with either the protonated or deprotonated N3 cytosines atoms, mimicking the experimental systems at pH 5.0 and 8.0 conditions, respectively. The simulations have been carried out for 150 ns, and the four trajectories have been analyzed in a comparative way. The root mean square deviation (RMSD), describing the evolution of the sampled conformations in terms of distance from the starting structure, has been monitored to follow the stability of the functional elements integrated on the cages at the two different pH values. At pH 5.0 the clamp-switch is stable for both the systems, being characterized by negligible deviations from the starting structure (data not shown), a result in line with the previous simulation of the isolated clamp-switch (32). At pH 8 a significant deviation from the starting structure is observed for the triple helix hosted on the LT-cage (Figure 2A, red line), but not on the T-cage (Figure 2A, black line). A significant deviation in the RMSD values of the two strands forming the double helix is also evident only on the triple helix integrated on the LT-cage (Figure 2B, red line) and not for the T-cage (Figure 2B, black line). This is confirmed also by the number of W-C and Hoogsteen hydrogen bonds (HBs) evaluated as a function of time. An average number of 8 HBs interactions is observed along the trajectory at pH 8.0 of the T-cage (Figure 3A, black lines) which are almost completely lost in the LT-cage (Figure 3A, red lines). A similar behaviour is observed monitoring the time-dependent evolution of the W-C HBs. An average number of 14 HBs is maintained at pH 8.0 for the T-cage (Figure 3B, black lines), indicating a high stability of the double-helix portion, whilst a strong reduction to around 7–8 is observed for the LT-cage (Figure 3B, red lines). These data indicate that at pH 8.0 in the LT-cage there is a strong impairment not only of the triple but also of the double helical region of the clamp-switch. In line, the distance of the corresponding base pair as a function of time for the Hoogsteen (Supplementary Figure S1A) and W-C bases (Supplementary Figure S1B) is larger in the LT- (red lines) than in T- (black lines) cage, respectively. The stability of the triple helices in the T- and LT-cages has been also probed calculating the clamp-switch interaction energy using the MM/GBSA approach. Analysis of three 50 ns trajectory time windows performed at pH 8.0, namely from 0–50, 50–100 and 100–150 ns, indicated quite constant energy values ranging from −60 to −70 kcal/mol for the triple helix of the T-cage, much lower than those observed for the LT-cage, where the energy values increase as a function of time from −45 to −15 kcal/mol (Figure 4A). It is interesting to note that the values observed in the last time window for the LT-cage are in line with those expected for the unfolding process of the triple helix, similar to the values observed for the isolated clamp at pH 8.0 (32). The buried surface area (BSA) calculated at pH 8.0 for the T-cage triple helix is about 1.4 times larger than that observed for the LT-cage (Figure 4B). These data indicate that the clamp-switch engineered over the cage scaffold, without using a spacer, retains the triple helical structure also at pH 8.0, locking the clamp-switch mechanism. The presence of the spacer restores the pH-dependent switching mechanism observed for the isolated clamp-switch (26). These results demonstrate a strong role of the cage scaffold in modulating the stability of the triple helices. The decoupling of the responsive functional elements, increasing their distance from the scaffold, is required to achieve a pH dependent opening/closing mechanism as observed in the isolated clamp-switch in solution (31,32).

### Synthesis and experimental characterization of the T- and LT-cages

**Figure 5.**
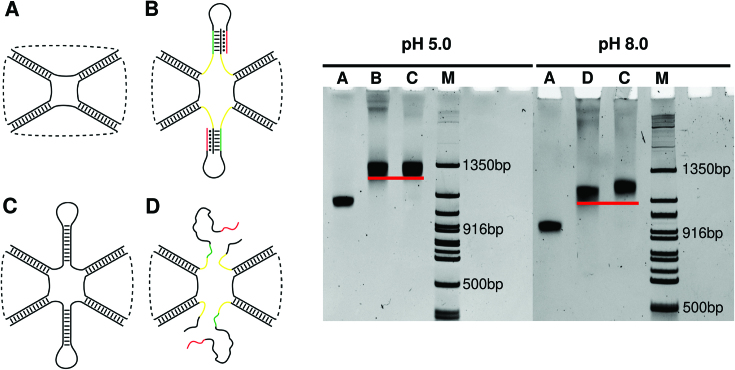
Gel-analysis of purified cages at pH 5.0 and 8.0. Lane M: DNA marker. Lane A: non-functionalized octahedral DNA cage, Lane B and D: LT-cage, Lane C: pH-independent cage.

The T- and LT-cage were assembled following validated protocols used for the assembly of other octahedral DNA structures published by our groups (20,53). The specific assembly of the two cages was tested analyzing the products obtained annealing an increasing number of equimolar amounts of the eight oligonucleotides, added one-by-one in successive order in a native polyacrylamide gel. As shown in the gel reported in Supplementary Figure S2, the presence of one well-defined product with higher molecular weight is observed in lane 8 upon mixing the cage-forming oligonucleotides, for both the T- (Supplementary Figure S2A) and LT-cage (Supplementary Figure S2B). The assembly efficiency is ∼40%, in agreement with previous reports of a DNA octahedral cage assembled without the presence of functional elements (11). The assembled cages have been analyzed by gel electrophoresis at pH 5.0 and 8.0 (Supplementary Figure S3B and Figure 5B) in comparison with a non-functionalized octahedral cage (Supplementary Figure S3A and Figure 5A) and to an octahedral cage engineered with two pH-independent motifs into one truncated face of the structure, mimicking the size of the clamp-switch (Supplementary Figure S3C and Figure 5C). The band of the T-cage, engineered with the pH-dependent motifs, runs at a height identical to the cage engineered with the pH-independent motifs, and both occur at a height different from the non-functionalized one, because of their higher molecular weight (Supplementary Figure S3). Interestingly, the T-cage runs identically either at pH 5.0 or at pH 8.0. This result confirms the outcomes from the MD simulations of the T-cage, indicating the lack of a pH-dependent clamp-switching behavior due to the stabilizing effect of the cage. Analysis of the LT-cage by gel electrophoresis at pH 5.0 and 8.0, indicates a noticeable pH dependent conformational change (Figure 5). The height of the band corresponding to the LT-cage runs identically to the cage engineered with the pH independent motifs at pH 5.0 but is characterized by a faster run at pH 8.0 (Figure 5B and C), confirming the need of a spacer to induce a pH-dependent conformational change.

### Probing the LT-cage pH-dependent behaviour by fluorescence spectroscopy

**Figure 6.**
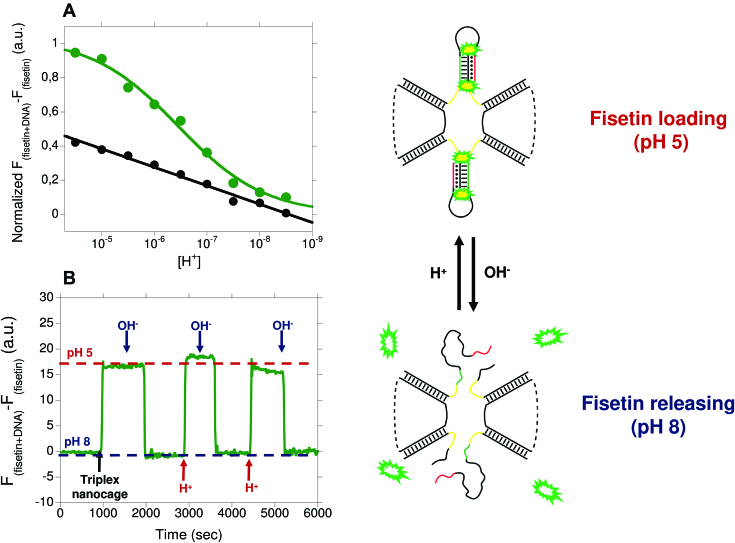
(**A**) pH−titration curves of the LT-cage (green curve) and its control duplex cage (black curve) achieved in TAE buffer at 25°C and using a cage concentration of 250 nM. Triplex-to-duplex transition is monitored taking the difference in the fisetin's fluorescence signal in presence and absence of the DNA cage at different pH values. (**B**) Reversible behaviour of the switching mechanism followed cyclically changing the pH of a solution containing the LT-cage and fisetin from 5.0 to 8.0.

To further demonstrate the pH-induced opening and closing mechanism of the clamp-switch in the LT-cage we have employed fisetin, a molecule that specifically binds triplex DNA through stacking interactions and, upon binding, enhances its fluorescence emission (54). This provides a convenient method to follow the opening/closing of the triplex clamp switch in the cage. We first tested the fisetin fluorescence behaviour and its selectivity towards triplex structure using, as a control, an isolated intramolecular triplex identical to that engineered in the cage (see supplementary data). The switch is composed by a double intramolecular hairpin stabilized with both W-C and parallel Hoogsteen interactions (Supplementary Figure S4). pH-titration curves at a fixed concentration of fisetin, show that its signal increases upon decreasing the pH (Supplementary Figure S5, red curve), due to its binding to the triplex. The observed pKa of the clamp intramolecular switch (p*K*_a_ = 5.6 ± 0.3) is in good agreement with previous studies of a similar switch (31). As a control, it has been observed that fisetin signal does not change over a wide pH range using a switch that can only form a duplex (Supplementary Figure S5, black curve). This further demonstrates the efficiency of fisetin for monitoring triplex formation. A similar approach has been used to monitor the pH-induced conformational change of the LT-cage. Of note, the observed p*K*_a_ of the triplex clamp–switch in the cage (Figure 6A, p*K*_a_ = 6.5 ± 0.3) is about 1 pH unit higher than that found with the isolated unimolecular triplex clamp-switch (p*K*_a_ = 5.6 ± 0.3). Finally, we demonstrate that the pH-induced conformational change of the LT-cage is reversible and rapid. The fisetin fluorescence change is consistent with the observed reversible opening/closing of the triplex clamp-switch in the LT-cage, as observed by cyclically changing the pH of the solution from pH 5.0 to 7.0 in the presence of fisetin (Figure 6B).

## DISCUSSION

The here reported results demonstrate the power of integrating experimental and simulative approaches for the rapid and correct design of functional, stimuli-responsive DNA nanostructures. We selected as a model system a truncated octahedral DNA nanostructure where a pH dependent clamp–switch triple helix has been engineered through a direct connection to the cage scaffold (T-cage, Figure 1C) or introducing a seven-thymidine spacer between the scaffold and the DNA triple-helix functional portion of the clamp-switch (LT-cage, Figure 1D). The behavior of triple helices as functional responsive elements has been studied in several contexts and purposes (25,55), such as for sensing solution pH (31,56), for directing chemical reactions (57), for strand displacement reactions (58–61) and for scaffolding of non-nucleic acid components (28,62), but this is the first time that the effect of nanostructure restrains on the formation and dissociation of a DNA triple helix has been investigated. A simulative screening of the structures indicated that only the functional elements hosted on the LT-cage show a pH dependent behavior, i.e. at pH 8.0 cytosine deprotonation induces a fast unfolding process of the triplex clamp switch (Figures 2–3). In the T-cage the clamp switching mechanism was not observed at pH 8.0. Evaluation of the interaction energy of the triple helix portion of the functional element through the MM/GBSA approach indicates for the T-cage values ranging from −70.0 to −60.0 kcal/mol, much lower than those observed for the LT-cage, ranging from −45.0 to −15.0 kcal/mol (Figure 4). This analysis indicates that the constraints imposed by the cage stabilize the triple helix by ∼30–40 kcal/mol hindering the pH dependent unfolding at high pH. The energies estimated through the MM/GBSA method do not always reproduce the experimental ones (63) but the energies calculated in this work, not in terms of absolute values but as difference between two distinct states, permit a reliable comparison, despite the limitations of the method. It is interesting to note that the nanostructure was able to induce similar constraints on the temperature dependent DNA hairpins previously engineered on the octahedral cage. These hairpins were able to undergo a temperature dependent unfolding process when investigated as isolated motif, but they could undergo only a deformation when engineered in the cage (12,22). In the case of the triple helix, the insertion of a seven-thymidine spacer between the functional element and the cage is essential to bypass the constraints and restore the pH-dependent switching properties of the isolated motif. Indeed, the interaction energy calculated for the triple helix in the last part of the simulation in the LT-cage (Figure 4A) is identical to that found for the isolated motif (32), in line with a complete unfolding of the clamp. The experimental results fully confirm the computational prediction. Only the LT-cage runs with a different mobility in an electrophoretic gel when brought at high pH values (Figure 5), a result confirmed following the fluorescence signal of the flavonoid fisetin as a function of pH (Figure 6). Fisetin is able to specifically bind only a fully formed triplex structure interacting with the two terminal regions (54), becoming fluorescent only upon binding to the DNA triplex. This permits to monitor the pH dependent switching mechanism simply following fisetin fluorescence intensity. Fluorescence experiments confirm the pH dependent mechanism of the LT-cage functional elements that is fully reversible working like the isolated clamp–switch in solution. This confirms that insertion of seven thymidine spacers decouples the functional element from the cage scaffold, preserving the structural/dynamic properties of the isolated motif. As a final comment we like to remark that user-friendly software like CadNano (64) and CanDo (65) are valuable tools for designing and predicting the assembly of static DNA structures, but they are unable in predicting the behavior of functional elements that can be studied by the use of MD techniques. The MD approach in fact allows to predict the effect of environmental conditions (like the effect of scaffold restrains), which represents a crucial aspect in engineering functional DNA nanostructures. This approach can be extended to any other non-canonical DNA structures or to more complex functional elements, like aptamers or antibodies, to be integrated on every kind of DNA scaffold ranging from small DNA tiles to large-sized DNA origami.

## Supplementary Material

Supplementary Data
