# Supplementary material for: Engineering a responsive DNA triple helix into an octahedral DNA nanostructure for a reversible opening/closing switching mechanism: a computational and experimental integrated study
Source: Nucleic Acids Res. 2018 Sep 22;46(19):9951–9. doi: 10.1093/nar/gky857 (PMC6212788; doi:10.1093/nar/gky857)
Supplement: Supplementary Data [file gky857_supplemental_files.pdf]

SUPPLEMENTARY DATA

Engineering a DNA triple helix into an octahedral DNA nanostructure for a reversible opening/closing switching mechanism: a computational and experimental integrated study

Alessio Ottaviani[1†], Federico Iacovelli[1†], Andrea Idili[2], Mattia Falconi[1], Francesco Ricci[2*] and Alessandro Desideri[1*]

[1] Biology Department, University of Rome Tor Vergata, Rome, 00133, Italy
[2] Chemistry Department, University of Rome Tor Vergata, Rome, 00133, Italy

[†] These authors contributed equally
* Co-corresponding authors

Oligonucleotides Section

T-cage

**O1**	*CCTCTTT*CCTTCTCTCGTTTGCTCTCTTCCT**TTCTCC**TCGGACCGTGATTCCATGACCTTAGAGTTGCCACCAGGTTTTTCGATGTCTAAGCTGACCGTTTTTTTC**GGAGAA**
**O2**	TGGCTACAGTTTTTCGGTCAGCTTAGACATCGTTTTTGAATCCTATGCTCGGACGTTTTTGGCTCACAT
**O3**	TCACGGTCCTTTTTCTATCCGATCGAGGCATGTTTTTCATACTGAGAGCGTTCCGTTTTTGTCATGGAA
**O4**	*CCTCTTT*CCTTCTCTCGTTTGCTCTCTTCCT**TTCTCC**TCCTGTAGCCAATGTGAGCCTGTCGCAGTTCAGATACGCTTTTTCATGCCTCGATCGGATAGTTTTTTTC**GGAGAA**
**O5**	CTCAGTATGTTTTTCGGTTACGGTACAATGCCTTTTTCGCAAGACGTTAGTGTCCTTTTTCGGAACGCT
**O6**	GGTGTATCGTTTTTGGCATTGTACCGTAACCGTTTTTGCGTATCTGAACTGCGACTTTTTCCACCGAAT
**O7**	CGTCTTGCGTTTTTGTATGACGCAGCACTTGCTTTTTCCTGGTGGCAACTCTAAGTTTTTGGACACTAA
**O8**	ATAGGATTCTTTTTGCAAGTGCTGCGTCATACTTTTTCGATACACCATTCGGTGGTTTTTCGTCCGAGC

LT-cage

**O1**	*CCTCTT*TCCTTCTCTCGTTTGCTCTCTTCC*T***TTCTCC**TCTTTTTTTGGACCGTGATTCCATGACTTTTTTCTTAGAGTTGCCACCAGGTTTTTCGATGTCTAAGCTGACCGTTTTTTTTC**GGAGAA**
**O2**	TGGCTACAGTTTTTCGGTCAGCTTAGACATCGTTTTTGAATCCTATGCTCGGACGTTTTTGGCTCACAT
**O3**	TCACGGTCCTTTTTCTATCCGATCGAGGCATGTTTTTCATACTGAGAGCGTTCCGTTTTTGTCATGGAA
**O4**	*CCTCTTT*CCTTCTCTCGTTTGCTCTCTTCCT**TTCTCC**TCTTTTTTTCTGTAGCCAATGTGAGCCTTTTTTGTCGCAGTTCAGATACGCTTTTTCATGCCTCGATCGGATAGTTTTTTTTC**GGAGAA**
**O5**	CTCAGTATGTTTTTCGGTTACGGTACAATGCCTTTTTCGCAAGACGTTAGTGTCCTTTTTCGGAACGCT
**O6**	GGTGTATCGTTTTTGGCATTGTACCGTAACCGTTTTTGCGTATCTGAACTGCGACTTTTTCCACCGAAT
**O7**	CGTCTTGCGTTTTTGTATGACGCAGCACTTGCTTTTTCCTGGTGGCAACTCTAAGTTTTTGGACACTAA
**O8**	ATAGGATTCTTTTTGCAAGTGCTGCGTCATACTTTTTCGATACACCATTCGGTGGTTTTTCGTCCGAGC

For the above sequences, the bases in bold represent the duplex forming portion of the switch. The underlined bases represent the random loop sequences and the italic bases represent the triplex forming portion. Red characters indicate the seven-thymidine spacers.

pH-independent cage

**O1**	GCCACCAGGTTTTTCGATGTCTAAGCTGACCGTCGAATATTTCCCCCCCCCCAGAAACCTTCCTGGACCGTGATTCCATGACTTTTTCTTAGAGTT
**O2**	TGGCTACAGTTTTTCGGTCAGCTTAGACATCGTTTTTGAATCCTATGCTCGGACGTTTTTGGCTCACAT
**O3**	TCACGGTCCTTTTTCTATCCGATCGAGGCATGTTTTTCATACTGAGAGCGTTCCGTTTTTGTCATGGAA
**O4**	CAGATACGCTTTTTCATGCCTCGATCGGATAGTCGAATATTTCCCCCCCCCCAGAAACCTTCCTCTGTAGCCAATGTGAGCCTTTTTGTCGCAGTT
**O5**	CTCAGTATGTTTTTCGGTTACGGTACAATGCCTTTTTCGCAAGACGTTAGTGTCCTTTTTCGGAACGCT
**O6**	GGTGTATCGTTTTTGGCATTGTACCGTAACCGTTTTTGCGTATCTGAACTGCGACTTTTTCCACCGAAT
**O7**	CGTCTTGCGTTTTTGTATGACGCAGCACTTGCTTTTTCCTGGTGGCAACTCTAAGTTTTTGGACACTAA
**O8**	ATAGGATTCTTTTTGCAAGTGCTGCGTCATACTTTTTCGATACACCATTCGGTGGTTTTTCGTCCGAGC

Non-functionalized octahedral cage

**OL1**	GCCACCAGGTTTTTCGATGTCTAAGCTGACCGTTTTTGGACCGTGATTCCATGACTTTTTCTTAGAGTT
**OL2**	TGGCTACAGTTTTTCGGTCAGCTTAGACATCGTTTTTGAATCCTATGCTCGGACGTTTTTGGCTCACAT
**OL3**	TCACGGTCCTTTTTCTATCCGATCGAGGCATGTTTTTCATACTGAGAGCGTTCCGTTTTTGTCATGGAA
**OL4**	CAGATACGCTTTTTCATGCCTCGATCGGATAGTTTTTCTGTAGCCAATGTGAGCCTTTTTGTCGCAGTT
**OL5**	CTCAGTATGTTTTTCGGTTACGGTACAATGCCTTTTTCGCAAGACGTTAGTGTCCTTTTTCGGAACGCT
**OL6**	GGTGTATCGTTTTTGGCATTGTACCGTAACCGTTTTTGCGTATCTGAACTGCGACTTTTTCCACCGAAT
**OL7**	CGTCTTGCGTTTTTGTATGACGCAGCACTTGCTTTTTCCTGGTGGCAACTCTAAGTTTTTGGACACTAA
**OL8**	ATAGGATTCTTTTTGCAAGTGCTGCGTCATACTTTTTCGATACACCATTCGGTGGTTTTTCGTCCGAGC

Unimolecular clamp-switch

CCTCTT-TCCTTCTCTCGTTTGCTCTCTTCCT-**TTCTCC**-TTTT-**GGAGAA**

Control duplex

TTCTCC-TTTT-**GGAGAA**

For the above sequences, the bases in bold represent the duplex forming portion. In the clamp-switch, the underlined bases represent the random loop sequences and the italic bases represent the triplex forming portion.

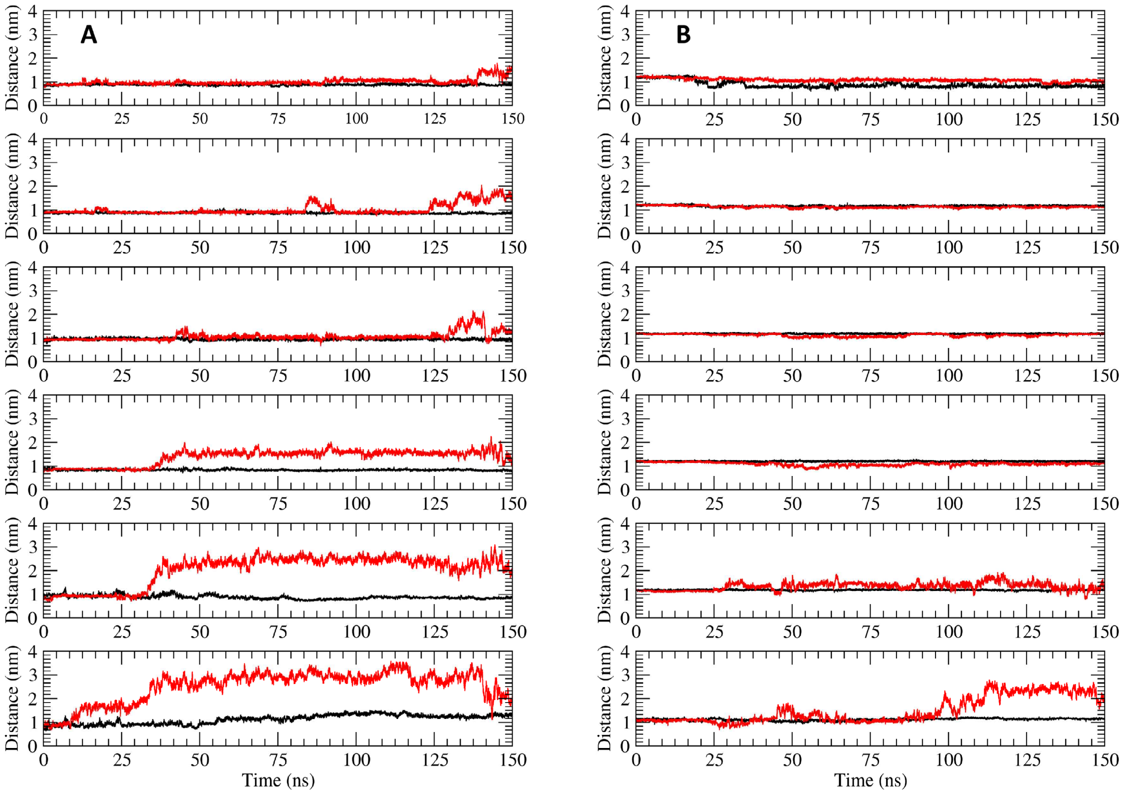

Figure S1. Time dependent evolution of base pairs distances**.** (A) Time dependence of the distance between the mass centers of the base pairs involved in the Hoogsteen interactions at pH 8.0 for the T- (black line) and LT-cage (red line). B) Time dependence of the distance between the mass centers of the base pairs involved in W-C HBs at pH 8.0 for the T- (black line) and LT-cage (red line).

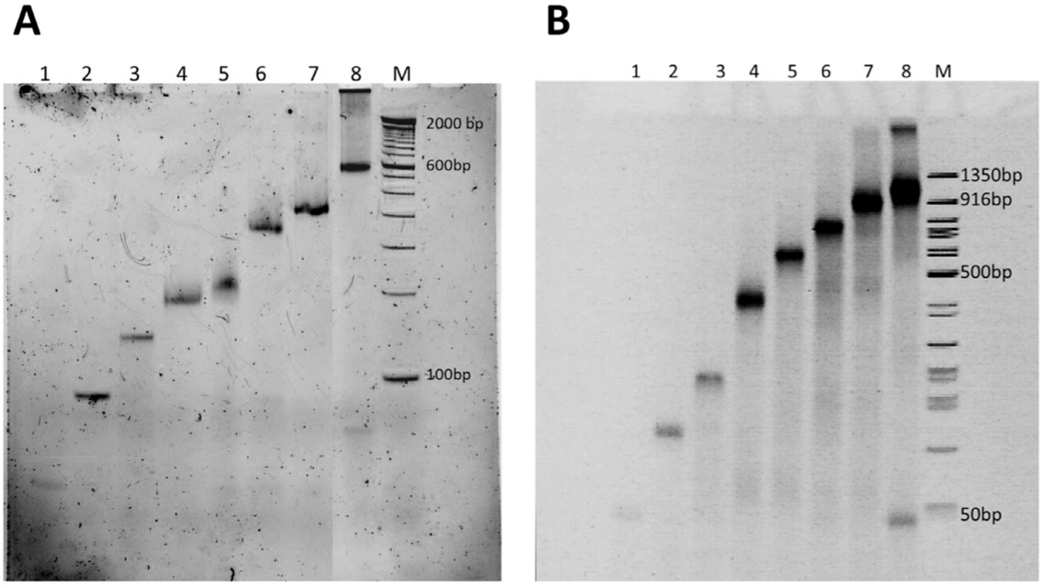

Figure S2. Gel-electrophoretic analyses of the cage assembly**.** Ladder experiments obtained increasing the numbers of DNA oligonucleotides that form the T (A) and LT (B) cages Lane M: DNA marker. Lanes 2-9 show the results of the assemby, increasing the number of oligonucleotides (i.e. O1; O1-O2; O1-O2-O3; O1-O2-O3-O4; O1-O2-O3-O4-O5; O1-O2-O3-O4-O5-O6; O1-O2-O3-O4-O5-O6-O7; O1-O2-O3-O4-O5-O6-O7-O8).

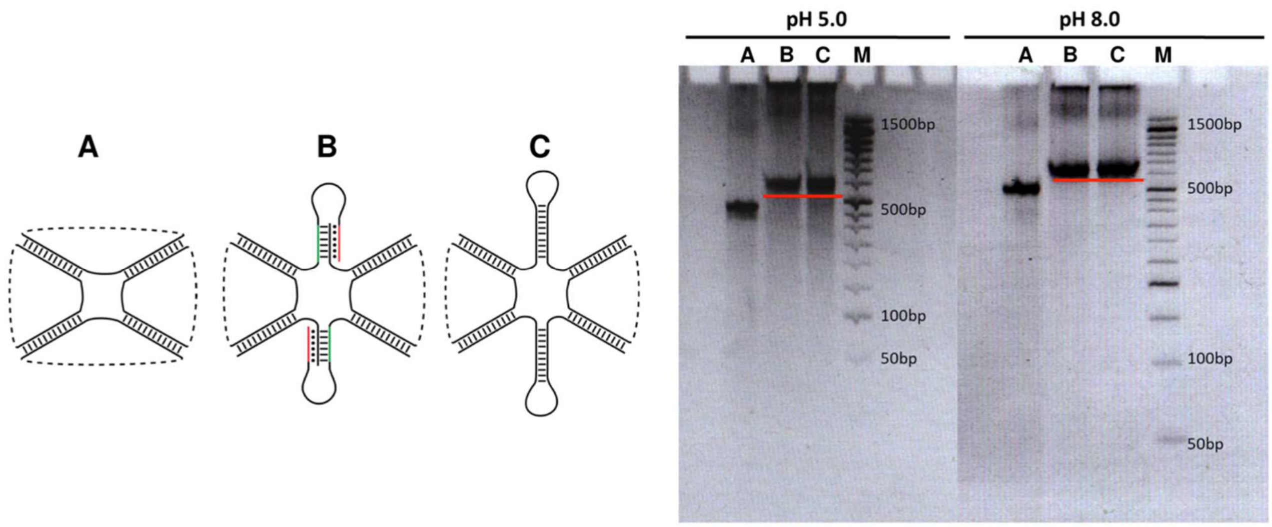

Figure S3. Gel-analysis of purified cages at pH 5.0 and 8.0. Lane M: DNA marker. Lane A: non-functionalized octahedral DNA cage, Lane B: T-cage, Lane C: pH-independent cage.

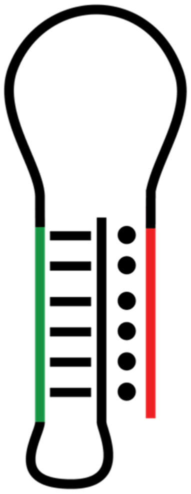

Figure S4. The isolated switch is composed by an intramolecular hairpin formed by the W-C hybridization of two six- base complementary sequences (black and green strands connected by thick lines), separated by a five bases loop. The duplex DNA is able to form a triplex structure via the formation of a second hairpin through Hoogsteen parallel interactions with the triplex forming sequence of the switch (red and black strands connected by dashes).

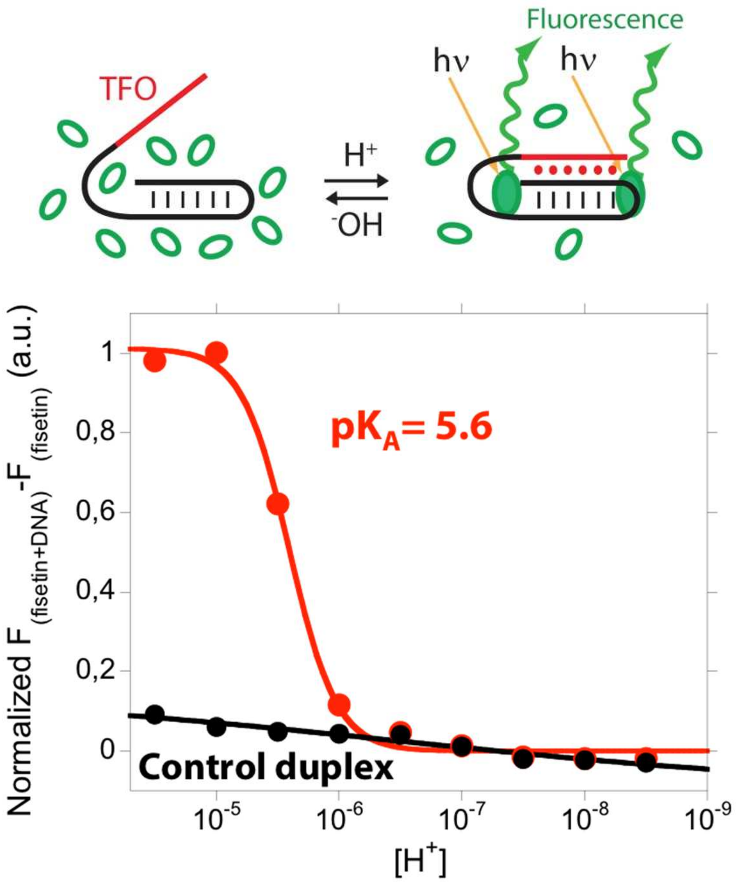

Figure S5. pH curves of the clamp triplex (red) and control duplex (black) by measuring the fluorescence fisetin signal at different pH values (see experimental section for details).
